# Storage Stability of a Multifunctional Fermented Blend Based on Sacha Inchi (*Plukenetia volubilis*) Oil Press Cake and Yacon (*Smallanthus sonchifolius*) Flour: Physicochemical Properties, Bioactivity, and Prebiotic–Probiotic Potential

**DOI:** 10.3390/foods15122131

**Published:** 2026-06-13

**Authors:** David Campos, Rosana Chirinos, Ana Aguilar-Galvez, María P. Carrasco, Romina Pedreschi

**Affiliations:** 1 Instituto de Biotecnología, Universidad Nacional Agraria La Molina, Av. La Molina s/n, Lima 12056, Peru; chiri@lamolina.edu.pe (R.C.); aaguilar@lamolina.edu.pe (A.A.-G.); pia.carrasco89@gmail.com (M.P.C.); 2 Research Group on Functional Foods and Nutraceuticals, Universidad Nacional Agraria La Molina, Av. La Molina s/n, Lima 12056, Peru; 3 Escuela de Agronomía, Pontificia Universidad Católica de Valparaíso (PUCV), Calle San Francisco s/n, La Palma, Quillota 2260000, Chile; romina.pedreschi@pucv.cl; 4 Millennium Institute Center for Genome Regulation (CRG), Santiago 8320000, Chile

**Keywords:** agro-industrial by-products, fructooligosaccharides (FOS), enzymatic hydrolysis, fermentation, storage stability, biofunctional properties

## Abstract

Plant-based symbiotic systems are often limited by poor storage stability and inconsistent biofunctional performance. This study evaluated the stability and functionality of a fermented blend based on sacha inchi (*Plukenetia volubilis*) oil press cake (SIC) and yacon (*Smallanthus sonchifolius*) flour (YF) as sources of protein and fructooligosaccharides (FOS), respectively, using two processing strategies: fermentation with *Lactobacillus rhamnosus* (T1) and combined enzymatic hydrolysis with Alcalase and fermentation with *Lactobacillus plantarum* (T2). Both treatments maintained viable cell counts (VCC) above probiotic thresholds (>10^6^ CFU mL^−1^) during 28 days of storage at 4 °C, confirming their suitability as probiotic carriers. Notably, T2 significantly enhanced metabolic activity, as evidenced by higher organic acid production and increased soluble protein content due to Alcalase-mediated hydrolysis, which promoted the generation of bioactive peptides associated with improved antioxidant and antihypertensive activities. Biofunctional properties, including total phenolic content, antioxidant capacity (AC), and angiotensin-converting enzyme (ACE) inhibitory activity, remained stable throughout storage, while FOS degradation was minimal, confirming preservation of prebiotic functionality. LC–MS/MS Q-TOF analysis revealed a complex phenolic profile that was differentially modulated by lactic acid fermentation, with *L. plantarum* (T2) promoting extensive phenolic biotransformation and increased metabolite diversity, whereas *L. rhamnosus* (T1) largely preserved the original phenolic profile. These findings demonstrate that the synergistic interaction between enzymatic hydrolysis and *L. plantarum* fermentation promoted peptide release, intensified microbial metabolism, and enhanced phenolic biotransformation, thereby contributing to the superior functional properties observed in T2, while maintaining stable biofunctional characteristics throughout refrigerated storage in both treatments.

## 1. Introduction

In recent years, increasing attention has been directed toward the development of functional foods that provide health benefits beyond basic nutrition, owing to their content of bioactive compounds capable of modulating physiological processes, improving gut health, and preventing chronic diseases, including cardiovascular and metabolic disorders [1]. Within this context, fermented products enriched with bioactive compounds, probiotics, and prebiotics have emerged as promising dietary strategies due to their potential to enhance intestinal health and reduce disease risk. Fermentation not only improves the nutritional profile of food matrices but also promotes the release of bioactive peptides, phenolic compounds, and other metabolites exhibiting antioxidant, hypoglycemic, antihypertensive, and anti-inflammatory activities [2,3,4].

Agro-industrial by-products represent a sustainable and high-value source of functional ingredients, enabling waste valorization and reducing the environmental impact associated with the food industry. Among them, sacha inchi (*Plukenetia volubilis*) oil press cake is a protein-rich by-product generated during oil extraction, characterized by its high content of essential amino acids, such as lysine, histidine, and leucine, and contains important amounts of isoleucine, valine, tryptophan, and phenylalanine and low concentrations of threonine and methionine [5,6] and its potential to produce antioxidant and antihypertensive peptides through enzymatic hydrolysis [7,8] and fermentation processes [9]. Similarly, yacon (*Smallanthus sonchifolius*) is recognized as an important source of FOS, which are associated with prebiotic activity and modulation of the gut microbiota. In addition, yacon contains phenolic compounds, mainly hydroxycinnamic acid derivatives, that contribute to its antioxidant potential [10]. Recent studies have highlighted lactic acid bacteria (LAB) fermentation as a sustainable strategy for the valorization of plant-based by-products, thereby enabling the development of functional foods enriched with bioactive compounds. LAB fermentation has also been associated with improved stability and bioaccessibility of phenolic compounds, enhanced antioxidant activity, and the generation of bioactive peptides and prebiotic carbohydrates in plant-based systems during storage. Importantly, microbial fermentation has proven effective in converting low-value by-products into high-value functional ingredients [11]. Similarly, recent reviews have emphasized that the fermentation of agro-industrial by-products represents a sustainable approach for producing value-added functional beverages with enhanced bioactive potential and consumer appeal [12].

Current studies have also highlighted growing interest in plant-based fermented beverages; however, research has primarily focused on conventional substrates such as soy and rice. Expanding the range of raw materials, including legumes, nuts, cereals, pseudocereals, and seeds, represents a promising approach to enhance nutritional and functional properties.

In this context, combining substrates rich in proteins, polyphenols, and prebiotic compounds represents a promising strategy for developing multifunctional fermented foods with probiotic and prebiotic potential. Fermentation enhances nutrient bioavailability and promotes the release of bioactive compounds, contributing to improved antioxidant activity and other health-related benefits [2,3]. For instance, Campos et al. [9] investigated the fermentation of a blend of SIC cake and YF, with *L. plantarum* and *L. rhamnosus*. Both strains exhibited adequate fermentation performance and comparable functional responses, whereas enzymatic hydrolysis significantly enhanced antioxidant and ACE inhibitory activities, with the simultaneous protease-assisted fermentation treatment showing the best overall performance. In that study, the optimization of the SIC:YF proportion was carried out using *L. plantarum*. The optimized formulation showed high probiotic viability, antioxidant capacity, ACE inhibitory activity, protein, dietary fiber, FOS, and short-chain fatty acid contents, highlighting its potential as a multifunctional fermented food.

Although previous studies have demonstrated the potential of enzymatic hydrolysis and lactic fermentation independently, limited information is available regarding their synergistic effects on the stability and functionality of plant-based symbiotic systems during storage. Therefore, we hypothesized that the synergistic interaction between Alcalase-mediated proteolysis and *L. plantarum* fermentation would enhance the generation and stability of bioactive compounds, thereby improving antioxidant capacity, ACE inhibitory activity, and overall functional stability during refrigerated storage. Therefore, this study aimed to evaluate the storage stability of a multifunctional fermented mixture based on SIC and YF after two treatments: fermentation with *Lactobacillus rhamnosus* and fermentation with *Lactobacillus plantarum* combined with protease hydrolysis.

## 2. Materials and Methods

### 2.1. Chemicals and Raw Materials

Glucose, fructose, sucrose, inulinase (I6285), Folin–Ciocalteu reagent (2 N), 2,2′-azinobis (3-ethylbenzothiazoline-6-sulfonic acid) (ABTS), and formic acid were purchased from Sigma-Aldrich (St. Louis, MO, USA). Lactic and acetic acid standards were obtained from Supelco (Bellefonte, PA, USA). HPLC-grade acetonitrile and methanol (LiChrosolv^®^), as well as Man, Rogosa, and Sharpe (MRS) culture medium, were purchased from Merck (Darmstadt, Germany). Alcalase^®^ 2.4 L was supplied by Novozymes (Bagsværd, Denmark). All other chemicals and solvents were of analytical grade and obtained from standard commercial suppliers.

SIC, obtained by cold pressing, was purchased from Olivos del Sur (Lima, Peru) local company in Lima, Peru. YF was produced from roots acquired at a local market in the Junín region, Peru, and processed into flour as previously described by Campos et al. [13]. The proximate chemical composition, sugar profile (glucose, fructose, and sucrose), FOS content, and soluble and insoluble dietary fiber contents of SIC and YF are presented in the Supplementary Material.

### 2.2. Experimental Setup

#### 2.2.1. Inoculum and Fermentation Preparation

**Figure 1:**
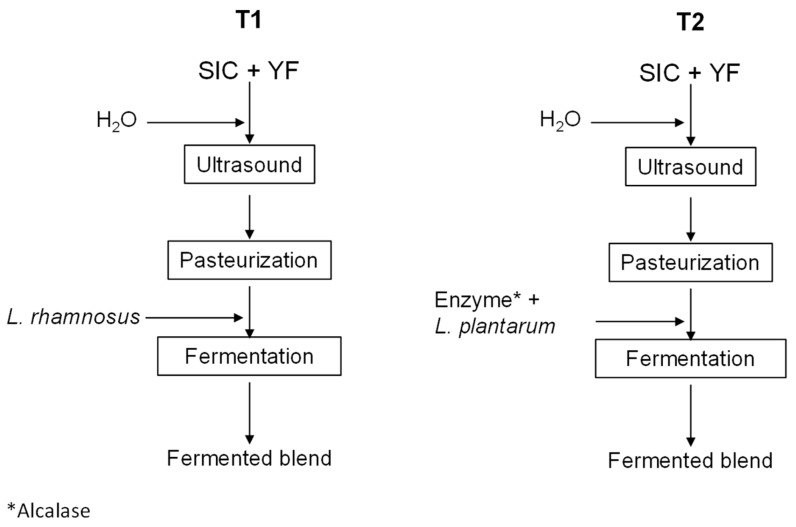
Schematic representation of fermentation treatments (T1 and T2) for the SIC–YF mixture.

The SIC and YF mixture fermentation was carried out using two bacterial strains: *Lactobacillus plantarum* NRRL B-3058 and *Lactobacillus rhamnosus* NRRL B-1937. The inoculum was prepared in 150 mL Erlenmeyer flasks containing 50 mL of MRS broth and incubated at 37 °C for 10 h in an orbital shaker at 125 rpm. The fermented SIC–YF mixtures were prepared according to the treatments described in Figure 1. For both treatments, the mixtures were pretreated by ultrasound using a Branson Ultrasonics SFX250 system (Branson Ultrasonics Corporation, Danbury, CT, USA), equipped with a 20 kHz converter and a ½″ microtip, operated at 40% amplitude for 5 min using 5 s pulse cycles (on/off). Pasteurization was subsequently performed at 95 °C for 10 min, followed by rapid cooling and inoculation at 1% (*v*/*v*) of the prepared inoculum (7 Log CFU mL^−1^). Fermentation was carried out in 250 mL flasks containing 50 mL of medium at 37 °C in a shaking incubator (SI-300, Lab Companion series, Jeio Tech, Daejeon, Republic of Korea) at 125 rpm for 13 h.

In treatment T1, after ultrasound pretreatment and pasteurization, *L. rhamnosus* NRRL B-1937 was used as the starter culture. In contrast, treatment T2 involved simultaneous protein hydrolysis with Alcalase^®^ (2 µL g^−1^ protein) and fermentation with *L. plantarum* NRRL B-3058. The concentrations of SIC and YF were selected based on previous studies and were 9.5% and 9.8% for *L. rhamnosus* (Supplementary Material) and 10.0% and 8.3% for *L. plantarum* [9], respectively.

#### 2.2.2. Stability During Storage

The stability of the fermented mixtures was evaluated over 28 days at 4 °C, simulating refrigerated storage conditions. Analyses were performed on days 0, 7, 14, 21, and 28. The following parameters were determined: VCC, pH, titratable acidity (TA), total and soluble protein content, FOS content and profile, lactic and acetic acid concentrations, dietary fiber, soluble and total solids, sugar content (glucose, fructose, and sucrose), total phenolic content and profile, AC (ORAC assay), and ACE inhibitory activity (IC_50_ value). These parameters were selected to comprehensively assess the physicochemical, microbiological, and biofunctional stability of the system.

### 2.3. Analytical Determinations

#### 2.3.1. Determination of pH and Titratable Acidity

The pH of fermented and non-fermented samples was measured directly, without prior sample preparation, using a calibrated pH meter with automatic temperature compensation, according to AOAC [14], at room temperature. TA was determined following the method described by Baba et al. [15], with slight modifications. Briefly, 5 g of the sample was homogenized with 25 mL of distilled water and filtered through Whatman filter paper. An aliquot of the filtrate was mixed with three drops of phenolphthalein and titrated with 0.1 mol L^−1^ NaOH until a stable pale pink endpoint was maintained for 30 s. Results are expressed as a percentage of lactic acid equivalents (%).

#### 2.3.2. Viable Cell Count

A 1 mL aliquot of the fermented culture was serially diluted in 9 mL of sterile physiological saline solution. Then, 100 μL aliquots of appropriate dilutions were spread onto Petri dishes containing MRS agar. The plates were incubated at 37 °C for 48 h in an inverted position, and VCC were expressed as log CFU g^−1^.

#### 2.3.3. Analysis of Lactic and Acetic Acids

Lactic and acetic acids were quantified according to Aguilar-Galvez et al. [16]. Fermented samples (2 mL) were mixed with 4 mL of 5 mM H_2_SO_4_, centrifuged at 14,000× *g* for 30 min at 15 °C, and the supernatant was filtered through a 0.22 µm PTFE membrane filter. Aliquots (10 µL) were analyzed by HPLC (Waters 2695, Waters Corporation, Milford, MA, USA) equipped with an autosampler, a 996 photodiode array detector (PDA), and Empower 2 software, using an Aminex^®^ HPX-87H column (300 × 7.8 mm; Bio-Rad, Hercules, CA, USA) maintained at 60 °C. The mobile phase consisted of 5 mM H_2_SO_4_ at a flow rate of 0.6 mL min^−1^. Organic acids were identified and quantified based on retention times and external calibration using corresponding standards.

#### 2.3.4. Crude and Soluble Protein Analysis

Total protein content (%) was determined using the micro-Kjeldahl method according to AOAC 984.13 [12], applying a nitrogen-to-protein conversion factor of 5.7 as described by Sathe et al. [5].

Soluble protein content in fermented and non-fermented mixtures was determined following the method of Lowry et al. [17], with slight modifications. Samples (10 g) were centrifuged at 12,300× *g* for 15 min at 5 °C (Eppendorf 5430R, Hamburg, Germany). The pellet was washed with 10 mL of distilled water and centrifuged again under the same conditions. The combined supernatants were vacuum filtered (Whatman filter paper, 11 µm). Briefly, an aliquot of the extract (400 µL) was mixed with alkaline copper solution, followed by the addition of Folin–Ciocalteu reagent, and incubated in the dark. Absorbance was measured at 750 nm. Protein concentration was calculated using a bovine serum albumin (BSA) standard curve (0.5–3 mg mL^−1^), and results are expressed as mg g^−1^.

#### 2.3.5. Glucose, Fructose, Sucrose, and Fructooligosaccharide (FOS) Analysis

Fermented samples were centrifuged (12,411× *g*, 10 min, 20 °C; Eppendorf 5804R, Hamburg, Germany). Aliquots (10 mL) of fermented and non-fermented samples were mixed with 100 mL of 70% (*v*/*v*) ethanol in 250 mL flasks. The mixtures were heated at 100 °C in a water bath, cooled to room temperature, filtered, and the supernatants were collected. The residues were re-extracted under the same conditions using 75 mL of 70% (*v*/*v*) ethanol. The extracts were combined and concentrated under reduced pressure to a final volume of 20 mL. Aliquots were used for sugar quantification and FOS determination.

Glucose, fructose, and sucrose were quantified using an ACQUITY^®^ UPLC H-Class system (Waters Corp., Milford, MA, USA) equipped with a refractive index detector. Separation was carried out on a HILICpak VG-50 4E column (250 × 4.6 mm, 5 μm; Shodex, Showa Denko K.K., Tokyo, Japan) with a HILICpak VG-50G 4A guard column (4.6 × 10 mm). The mobile phase consisted of acetonitrile–methanol–water (75:20:5, *v*/*v*/*v*) at a flow rate of 0.8 mL min^−1^ at 60 °C. The injection volume was 5 μL. All solutions were filtered through 0.22 μm membrane filters (Millipore, Bedford, MA, USA) prior to analysis.

FOS were determined according to Jaime et al. [18] and Campos et al. [11], with slight modifications. Enzymatic hydrolysis was performed using inulinase. Briefly, 68 U of inulinase, dissolved in 0.5 mol L^−1^ sodium acetate buffer (pH 4.5), was added to 1.8 mL of extract. The mixture was incubated at 60 °C for 180 min. Released glucose and fructose were quantified as described above, and FOS content was calculated according to [19].

FOS profiling was performed using an ACQUITY UPLC H-Class system (Waters Corporation, Milford, MA, USA) coupled with an evaporative light scattering detector (ELSD). Separation was achieved by hydrophilic interaction liquid chromatography (HILIC) using a Shodex HILICpak VN-50 2D column (5 μm, 2.0 × 150 mm) with a HILICpak VN-50G 4A guard column (5 μm, 4.6 × 10 mm). A 5 μL aliquot of the extract was injected per run. ELSD conditions were as follows: drift tube temperature, 55 °C; carrier gas pressure, 379.2 kPa; gain, 50. Chromatographic separation was carried out at 55 °C with a flow rate of 0.3 mL min^−1^ under isocratic conditions using 80% acetonitrile as the mobile phase.

#### 2.3.6. Determination of Soluble Solids, Total Solids, and Dietary Fiber

A previously calibrated portable refractometer (ATAGO MASTER-53M, Tokyo, Japan, 0.0–53.0 °Brix) was used to determine soluble solids content (°Brix).

Moisture content was determined according to AOAC Method 934.01 [20]. Samples (3–5 g) were placed in pre-dried and weighed porcelain crucibles and dried at 100–105 °C until constant weight was achieved. After cooling in a desiccator, the samples were reweighed. Total solids were calculated from the weight difference and expressed as a percentage of the initial sample weight.

Dietary fiber was determined according to AOAC Method 991.43 [12], with slight modifications. This method is based on enzymatic digestion to separate and quantify soluble dietary fiber (SDF) and insoluble dietary fiber (IDF). A 1 g portion of the dehydrated samples was subjected to enzymatic digestion using a commercial kit (TDF100A, Sigma-Aldrich, USA). Residual protein and ash were subsequently determined for correction. Total dietary fiber (TDF) was calculated as the sum of SDF and IDF.

#### 2.3.7. In Vitro Antioxidant Capacity

AC was assessed using extracts prepared as described for soluble protein determination. The ORAC assay was performed in 96-well microplates (Brand, Wertheim, Germany) according to Ou et al. [21]. Each well received 25 μL of phosphate buffer (75 mM, pH 7.4; used as a blank), diluted sample, or Trolox standard and was incubated at 37 °C for 10 min with 250 μL of fluorescein (55 nM). The reaction was initiated by adding 25 μL of AAPH (153 nM), and fluorescence was monitored for 50 min at excitation and emission wavelengths of 485 and 528 nm, respectively, using a microplate reader (Synergy 2, BioTek, Winooski, VT, USA). Results are expressed as μmol Trolox equivalents (TE) g^−1^ of sample.

#### 2.3.8. Antihypertensive Activity

Angiotensin I-converting enzyme inhibitory activity was determined according to Wu et al. [22], with modifications by Chirinos et al. [23]. The assay was based on the enzymatic conversion of hippuryl–histidyl–leucine to hippuric acid (HA), which was quantified using an Acquity H-Class UPLC system (Waters, Milford, MA, USA) coupled with a photodiode array detector (PDA eλ detector, Waters, Milford, MA, USA). Briefly, 50 μL of 2.17 mM HHL solution, 10 μL of sample, and 10 μL of ACE solution (2 mU), both prepared in 100 mM borate buffer containing 300 mM NaCl (pH 8.3), were mixed and incubated at 37 °C for 30 min. The reaction was stopped by adding 80 μL of 1 N HCl. Samples were compared with a control and a blank subjected to the same reaction conditions. In the control, the 10 μL of sample was replaced with 10 μL of borate buffer, whereas in the blank, 10 μL of inactivated ACE solution (pretreated with 1 N HCl) was used. The reaction mixtures were filtered through a 0.22 μm Millipore GV filter (Millipore Sigma, Bedford, MA, USA). Subsequently, 2 μL of each sample was injected into a Kinetex C18 reversed-phase column (1.7 μm, 2.1 × 50 mm; Phenomenex, Torrance, CA, USA) equipped with a guard column. HA and HHL were detected at 228 nm. The mobile phase consisted of (A) 0.05% trifluoroacetic acid (TFA) in water and (B) 0.05% TFA in acetonitrile. The gradient program was as follows: 5% B from 0 to 1.5 min; 5–60% B over 2.5 min; 60–90% B over 0.5 min; maintained at 90% B for 1.5 min; returned from 90% to 5% B over 0.5 min; and finally equilibrated at 5% B for 1.5 min. The total run time was 8 min at 30 °C, with a flow rate of 0.5 mL min^−1^. ACE inhibition (%) was evaluated at protein concentrations ranging from 0.01 to 1.0 mg mL^−1^, and IC_50_ values were calculated from sigmoidal dose–response curves, defined as the protein concentration required to achieve 50% inhibition of ACE activity.

#### 2.3.9. Determination of Total Phenolic Content and Phenolic Profile

Total phenolic content (TPC) was assessed using extracts prepared as described for soluble protein determination. TPC was determined according to Singleton and Rossi [24] (1965), with slight modifications. The reaction mixture consisted of 500 µL of extract, 250 µL of Folin–Ciocalteu reagent (1 N), and 1250 µL of sodium carbonate solution (1.2 N). After incubation for 30 min in the dark, absorbance was measured at 755 nm. Results are expressed as mg gallic acid equivalents (GAE) g^−1^ or mL^−1^, as appropriate.

Phenolic compounds were identified using an ACQUITY UPLC I-Class system (Waters Corp., Milford, MA, USA) equipped with a BEH column (1.8 µm, 100 × 2.1 mm) and a guard column (1.7 µm, 5 × 2.1 mm), as previously described [25]. The system was coupled to a quadrupole time-of-flight mass spectrometer (Xevo G2-XS QTof; Waters Corp.) and a photodiode array detector (PDA). The mobile phases consisted of water with 0.1% formic acid (A) and acetonitrile with 0.1% formic acid (B). The gradient program was as follows: 2% B for 2 min, 2–7% B over 2 min, 7–12% B over 11 min, 12–26% B over 5 min, 26–55% B over 5 min, and 55–95% B over 1 min, followed by 100% B for 3 min and re-equilibration at 2% B for 3 min. The column temperature was maintained at 30 °C and the flow rate at 0.2 mL min^−1^.

Mass spectra were acquired using electrospray ionization (ESI) in negative mode (−3.5 kV), with a cone voltage of 40 V, source temperature of 120 °C, desolvation temperature of 550 °C, and desolvation gas flow of 1000 L h^−1^. Data were acquired in MS^e^ centroid mode with low collision energy (4 V) and a high-energy ramp (6–50 V). The instrument was calibrated with 0.5 M sodium formate, and leucine–enkephalin (200 pg µL^−1^; *m*/*z* 554.2615, ionization voltage 2.0 kV) was used as LockMass, infused at 10 µL min^−1^.

Chromatograms were processed using MassLynx v4.1 software (Waters Corp., Milford, MA, USA). Base peak ion (BPI) chromatograms were extracted at low and high energy for peak identification. The molecular ion [M–H]^−^ was obtained from low-energy spectra to determine elemental composition (mass error < 5 ppm), while high-energy spectra provided fragmentation patterns for compound identification based on comparison with databases and reference standards.

### 2.4. Statistical Analysis

All results are expressed as mean ± standard deviation (SD) of three independent replicates. Two-way analysis of variance (ANOVA), considering strain and storage time as fixed factors. When significant effects were detected (*p* < 0.05), mean comparisons between strains at each storage time were performed using Tukey’s honestly significant difference (HSD) test. Additionally, one-way ANOVA followed by Tukey’s HSD test was conducted separately for each strain to evaluate changes during storage. In the figures, different uppercase letters indicate significant differences between strains at the same storage time, whereas different lowercase letters indicate significant differences among storage times within the same strain (*p* < 0.05). All analyses were performed using Statgraphics^®^ Centurion XVI (StatPoint Technologies, Inc., Rockville, MD, USA).

## 3. Results

### 3.1. Acidification of the Medium and Cell Viability During Storage

**Figure 2:**
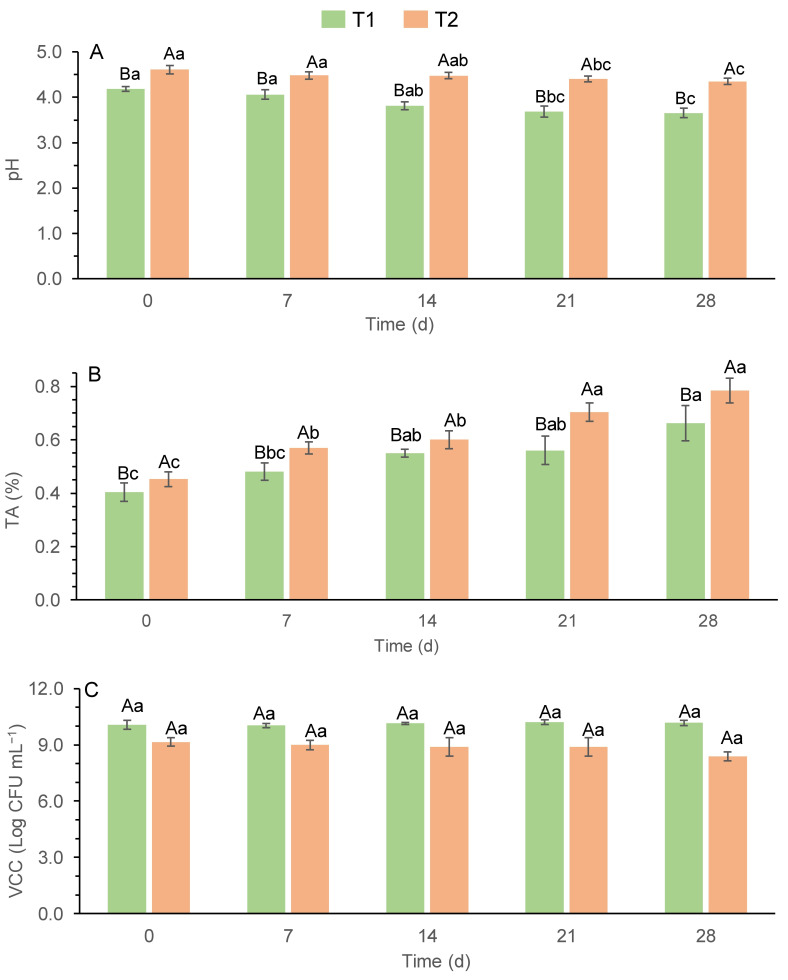
Evolution of pH (**A**), titratable acidity (**B**), and viable cell count (**C**) in a fermented mixture of sacha inchi and yacon flour during storage at 4 °C. Different uppercase letters indicate significant differences between strains at the same storage time, whereas different lowercase letters indicate significant differences among storage times within the same strain (*p* < 0.05).

The variations in pH, TA, and VCC are presented in Figure 2A–C. Two-way ANOVA showed that both strain and storage time significantly affected pH (*p* < 0.05). Additionally, a significant strain × time interaction was detected for pH, indicating distinct acidification patterns during storage. This effect could be related to T2, where fermentation occurred simultaneously with protein hydrolysis induced by Alcalase addition, which may have altered buffering capacity and acidification kinetics. For TA, both strain and storage time exerted significant effects (*p* < 0.05), whereas the strain × time interaction was not significant (*p* > 0.05), suggesting comparable trends during storage. In contrast, storage time significantly affected VCC (*p* < 0.05), whereas strain and the strain × time interaction were not significant (*p* > 0.05), indicating similar viability patterns during refrigerated storage.

Throughout storage, both treatments showed no significant changes (*p* > 0.05) in pH until day 14, after which a gradual decrease was observed. Overall, pH decreased during storage at 4 °C, with a significant reduction on day 28. The pH decreased by 0.56 units (from 4.19 to 3.66) and 0.26 units (from 4.61 to 4.35) for T1 and T2, respectively. Similarly, Shori et al. [26] reported a 0.3-unit decrease in pH in stored cashew milk-based yogurt fermented with probiotic *Lactobacillus* spp. strains. Furthermore, T1 showed significantly lower pH values (*p* < 0.05) than T2 throughout storage.

TA values increased gradually up to days 14 and 21 for T1 and T2, respectively. By day 28, TA had significantly increased (*p* < 0.05) from 0.40 to 0.66% in T1 and from 0.45 to 0.78% in T2. This increase may be attributed to bacterial metabolic activity supported by nutrient availability in the fermentation medium. SIC is rich in proteins, minerals, fiber, and phytochemicals, whereas YF provides sugars, FOS, and polyphenols. Thus, the observed decrease in pH and increase in TA are consistent with enhanced microbial metabolism. In T2, a smaller decrease in pH but a greater increase in TA was observed. This may be attributed to the higher degree of protein hydrolysis, which generates free terminal groups (–NH_2_ and –COOH) that enhance buffering capacity. Consequently, increased acidity does not necessarily result in a proportional decrease in pH. Additionally, the higher availability of low-molecular-weight peptides generated by Alcalase may have supported microbial metabolic activity during storage. TA values in plant-based fermented beverages typically range from 0.2% to 0.8%. Increased acidity may also contribute to improved antioxidant capacity by promoting the release and stabilization of bioactive compounds.

VCC results are shown in Figure 2C. At day 0, T1 reached 10.07 log CFU mL^−1^, whereas T2 showed 9.15 log CFU mL^−1^. VCC remained stable throughout storage, with no significant differences observed between treatments (*p* > 0.05). Although Alcalase-induced hydrolysis may have modified substrate composition, it did not negatively affect microbial viability in T2. This may be explained by the limited proteolytic activity of Alcalase under acidic conditions. Previous studies have reported variable trends in VCC depending on the matrix and strain used. VCC values in this study remained within the recommended range for probiotic products, with only a slight, non-significant reduction during storage to approximately 8.39 log CFU mL^−1^ in T2. These results indicate that the combined hydrolysis–fermentation process does not compromise microbial viability.

Overall, the fermented SIC–YF system effectively maintained the viability of *L. rhamnosus* and *L. plantarum* during storage, highlighting its potential as a probiotic carrier. This may be attributed to its composition, which supports microbial survival and activity. Notably, VCC values exceeded the minimum threshold (6–7 log CFU mL^−1^) required to ensure probiotic functionality.

### 3.2. Impact of Storage on Lactic Acid and Acetic Acid Contents

For lactic acid, a two-way ANOVA revealed that the strain, storage time, and their interaction significantly affected lactic acid production (*p* < 0.05). Tukey’s HSD test showed significant differences between treatments, with T2 exhibiting significantly higher lactic acid concentrations than T1. Similarly, for acetic acid, the two-way ANOVA showed the same trend, where strain, storage time, and their interaction significantly influenced acetic acid production (*p* < 0.05), with T2 presenting significantly higher concentrations than T1.

**Figure 3:**
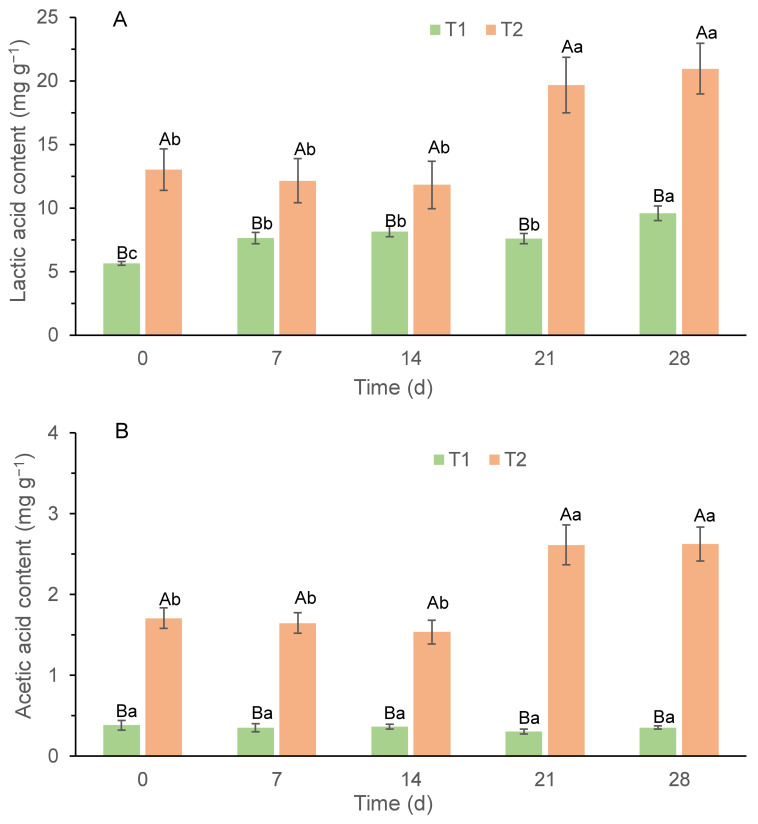
Lactic (**A**) and acetic acid (**B**) contents of fermented mixtures of sacha inchi cake and yacon flour during storage at 4 °C. Different uppercase letters indicate significant differences between strains at the same storage time, whereas different lowercase letters indicate significant differences among storage times within the same strain (*p* < 0.05).

The variations in lactic and acetic acid contents are presented in Figure 3A,B. Clear differences were observed between T1 and T2, attributable to the use of different starter cultures (*L. rhamnosus* in T1 and *L. plantarum* in T2) and substrate modifications resulting from Alcalase hydrolysis in T2. As suggested by [9], these differences may be associated with a higher concentration of low-molecular-weight peptides in T2, which could modulate microbial metabolism and organic acid synthesis.

Lactic acid content increased from 5.64 to 9.58 mg g^−1^ in T1 and from 13.01 to 20.96 mg g^−1^ in T2. A significant increase (*p* < 0.05) was observed on 28 days in T1 and at 21 days in T2. Coda et al. [27] reported a similar increase in lactic acid during storage of vegetable yogurt-like beverages and noted a stoichiometric relationship between sugar consumption and lactic acid formation. Likewise, Grasso et al. [28] found that plant-based yogurt-type products generally exhibit lower lactic acid content compared with dairy yogurt. In contrast, the values obtained in this study are comparable to those of dairy yogurt in T1 and higher in T2. This behavior is likely due to the high availability of fermentable sugars (glucose, fructose, and sucrose) and prebiotics (FOS) in the SIC–YF mixture, along with the presence of low-molecular-weight peptides in T2 [9], which may enhance microbial activity and organic acid production.

Regarding acetic acid (Figure 3B), a marked difference was observed between T1 and T2. In T1, acetic acid averaged 0.35 mg g^−1^ and remained stable throughout storage, whereas T2 showed an increase from 1.71 to 2.62 mg g^−1^, with a significant rise (*p* < 0.05) after 21 days. This divergence is consistent with Annunziata et al. [29], who reported that short-chain fatty acid (SCFA) biosynthesis is strongly influenced by substrate composition and microbial metabolism.

For prolonged storage, acetic acid levels in T2 were higher than those reported by Salmerón et al. [30] but closer to those reported by Küçükgöz et al. [31]. Lactic and acetic acids are the main products of lactic fermentation; however, acetic acid is associated with an unpleasant vinegar-like taste [30].

### 3.3. Changes in Soluble and Crude Protein During Storage

**Figure 4:**
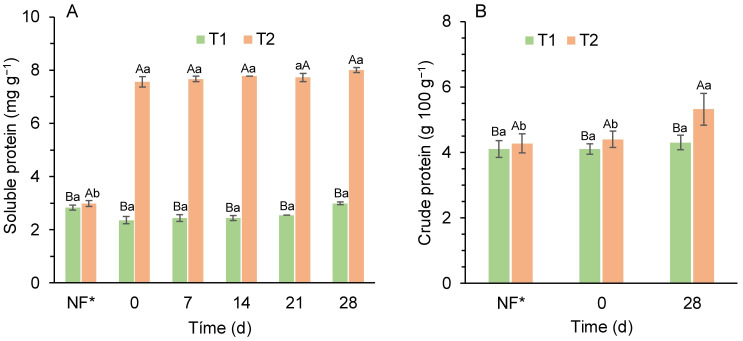
Soluble (**A**) and crude protein (**B**) contents of fermented and non-fermented (NF*) SIC and YF mixtures during storage. Different uppercase letters indicate significant differences between strains at the same storage time, whereas different lowercase letters indicate significant differences among storage times within the same strain (*p* < 0.05).

For soluble protein content, a two-way ANOVA revealed that strain, storage time, and their interaction significantly affected soluble protein levels (*p* < 0.05). Tukey’s HSD test demonstrated significant differences between treatments, with T2 exhibiting higher soluble protein concentrations than T1. Similarly, crude protein content was significantly influenced by strain, storage time, and their interaction (*p* < 0.05), and T2 showed significantly higher crude protein values than T1. The results for soluble and crude protein contents are shown in Figure 4A,B. Prior to fermentation, soluble protein levels were comparable between T1 and T2 (2.98 and 2.83 mg g^−1^, respectively). After fermentation, a clear divergence was observed: soluble protein decreased in T1 (2.36 mg g^−1^) but increased significantly in T2 (7.56 mg g^−1^; *p* < 0.05). This increase is attributed to Alcalase-mediated hydrolysis, which enhances protein breakdown and the release of low molecular weight peptides. In contrast, the reduction in T1 likely reflects microbial utilization of soluble nitrogen fractions. These findings are consistent with previous reports showing that enzymatic hydrolysis improves peptide solubilization and availability [32,33]. The lower values observed for T2 compared to those reported by Campos et al. [9], despite identical processing conditions, are likely attributable to differences in substrate composition, underscoring its key role in hydrolysis efficiency.

During storage, soluble protein levels in both T1 and T2 remained stable throughout the 28-day storage period. In T2, soluble protein levels were strongly influenced by the combined action of proteases produced by *L. plantarum* and the exogenous enzyme Alcalase, leading to extensive protein hydrolysis and enhanced solubilization, resulting in values approximately 2.7-fold higher than in T1. Similar effects have been reported in both dairy and plant-based systems treated with proteases. Soluble protein content in T1 was within the upper range typically reported for plant-based fermented products, whereas T2 greatly exceeded this range. The lower pH observed in T1 may have reduced protein solubility by approaching the isoelectric region of some protein fractions. In contrast, the enzymatic hydrolysis applied in T2 likely generated more hydrophilic low-molecular-weight peptides, which may have improved solubility and buffering capacity.

Crude protein content ranged from 4.10 to 5.32 g 100 g^−1^, with significant differences (*p* < 0.05) observed between treatments. In contrast, during storage, significant differences (*p* < 0.05) were only detected for T2 at day 28. Consistent with Deziderio et al. [34], fermentation did not significantly affect crude protein levels. Overall, T2 significantly increased soluble protein content without affecting crude protein levels, which may enhance digestibility and promote the generation of bioactive peptides [35]. In contrast, T1 showed limited changes, likely due to the lower proteolytic activity of *L. rhamnosus*. These results support the role of combined hydrolysis–fermentation strategies in improving protein functionality in plant-based systems.

### 3.4. Changes in Fructooligosaccharides, Sugars, Soluble and Total Solids, and Fiber During Storage

**Figure 5:**
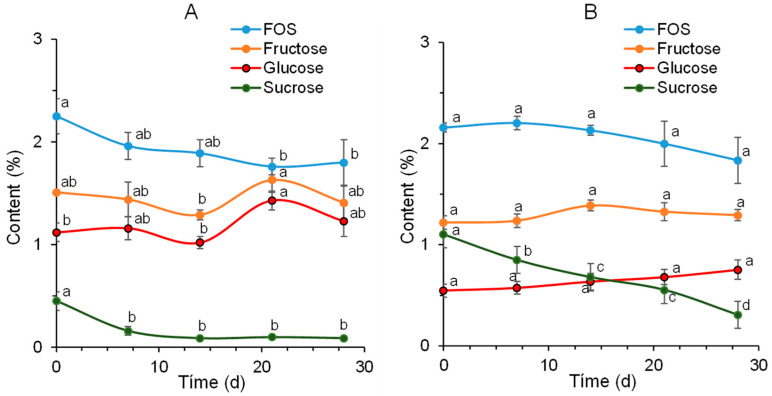
Changes in fructooligosaccharides, fructose, glucose, and sucrose during storage of fermented SIC–YF mixtures under treatments T1 (**A**) and T2 (**B**). Different letters among points within each curve indicate significant differences (*p* < 0.05, Tukey’s test).

Changes in FOS and sugars during storage are shown in Figure 5A,B. In both treatments, FOS remained the predominant carbohydrates throughout the 28-day storage period, although a slight progressive decrease was observed. This trend suggests partial hydrolysis of FOS into simpler sugars, mainly fructose and small amounts of glucose, likely associated with enzymatic activity and microbial metabolism of LAB present in the matrix.

In treatment T1, fermented with *L. rhamnosus* directly on the substrate, FOS levels remained relatively stable during most of the storage period, with a significant (*p* < 0.05) reduction observed toward the end (21–28 days). Treatment T2, fermented with *L. plantarum* with simultaneous protein hydrolysis, showed a similar pattern but with a slightly smaller decrease and no significant change (*p* > 0.05). This difference may be attributed to strain-specific metabolic characteristics and the enzymatic treatment applied in T2. The proteolytic action of Alcalase^®^ likely increased the availability of peptides and amino acids, which may have modulated microbial metabolism and reduced carbon demand during storage.

Simple sugars, particularly glucose and fructose, remained relatively stable during storage. In contrast, sucrose showed a marked decrease in both treatments, suggesting early hydrolysis during storage. Similar trends were reported by Chun et al. [33] in fermented sugarcane juice. These authors also reported that sucrose is more readily utilized by bacteria than prebiotic oligosaccharides, which is consistent with the results obtained in this study.

From a metabolic perspective, some strains of *Lactobacillus* possess β-fructosidases capable of hydrolyzing fructans such as inulin and FOS, releasing mainly fructose [36], and can also act on sucrose, yielding both fructose and glucose. In addition, LAB can metabolize fructans through specific transport systems and intracellular enzymes, facilitating their use as carbon sources and promoting their persistence in fermented matrices [37,38]. FOS metabolism may occur through two mechanisms: (i) intracellular transport followed by cytoplasmic hydrolysis, or (ii) extracellular hydrolysis mediated by cell-associated enzymes followed by uptake of released sugars [38]. The released fructose can subsequently enter glycolysis or alternative fermentative pathways, contributing to metabolite formation in LAB. These metabolic pathways support the utilization of complex fructans in plant-based matrices.

**Figure 6:**
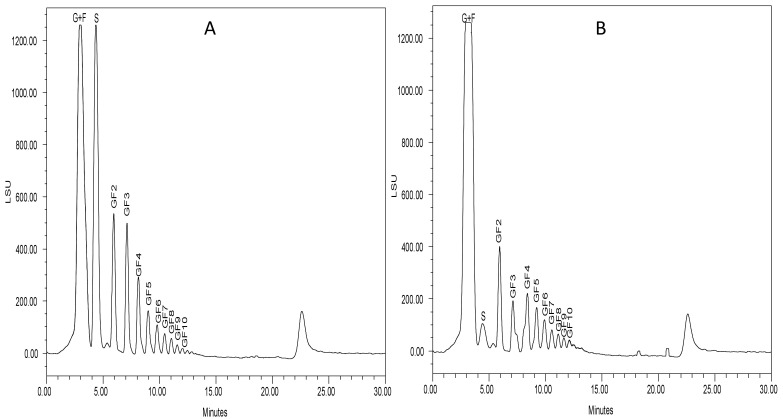
Fructooligosaccharide profile obtained from treatment T2 on day 0 of storage (**A**) and after 28 days of storage (**B**). F, fructose; G, glucose; S, sucrose; FOS, (GF2–GF10)

Figure 6 shows the FOS profile determined by HILIC–ELSD in samples corresponding to treatment T2 at day 0 and day 28 of storage. The chromatograms exhibit a high degree of similarity in the number of detected components, including glucose + fructose, sucrose, kestose (GF2), nystose (GF3), and oligomers with a higher degree of polymerization (GF4–GF10). Although variations in relative peak areas were observed, no changes in the number of compounds detected were evidenced, suggesting qualitative stability of the FOS profile during storage. A comparable trend was observed for treatment T1.

From a functional perspective, the limited reduction in FOS during storage indicates that the fermented matrix retained a substantial fraction of carbohydrates with prebiotic potential. The stability of these compounds is critical for maintaining functionality in fermented foods enriched with prebiotics. Previous studies have highlighted that prebiotic stability during processing and storage is essential for preserving their biofunctionality [36]. Overall, these results indicate that fermentation produced a matrix with a relatively stable prebiotic fraction, supporting its potential application as a functional ingredient in value-added food products.

Table 1 presents the evolution of soluble solids, total solids, and dietary fiber composition in fermented SIC–YF blends during 28 days of refrigerated storage. No significant differences (*p* > 0.05) were observed in soluble or total solids between treatments and during storage, indicating high physicochemical stability of the system. Soluble solids remained relatively constant, with a slight decrease in T1 (from 7.63 to 7.40 °Brix) and minimal variation in T2, suggesting limited post-fermentation metabolic activity and good stability during storage. These values are consistent with those reported by Deziderio et al. [32] for fermented plant-based products (1–4 °Brix) and fermented milk (~7 °Brix).

Regarding dietary fiber, both treatments maintained stable levels throughout storage. Soluble fiber showed a slight increase in T2 (from 0.87 to 0.99%), whereas insoluble fiber remained unchanged in both treatments, indicating that fermentation and storage did not significantly affect structural polysaccharides. Total dietary fiber remained consistent, with a slight increase in T2 on day 28 (3.59%). This preservation of fiber fractions is particularly relevant, as it supports the retention of prebiotic functionality and suggests that the fermentation process does not compromise the structural integrity of fiber components. Overall, these results demonstrate that the combined hydrolysis–fermentation strategy preserves key physicochemical and functional properties during storage, reinforcing the suitability of the system as a stable plant-based functional ingredient.

### 3.5. Evaluation of the Stability of Phenolic Compounds During Storage and Identification of Phenolic Compounds

**Figure 7:**
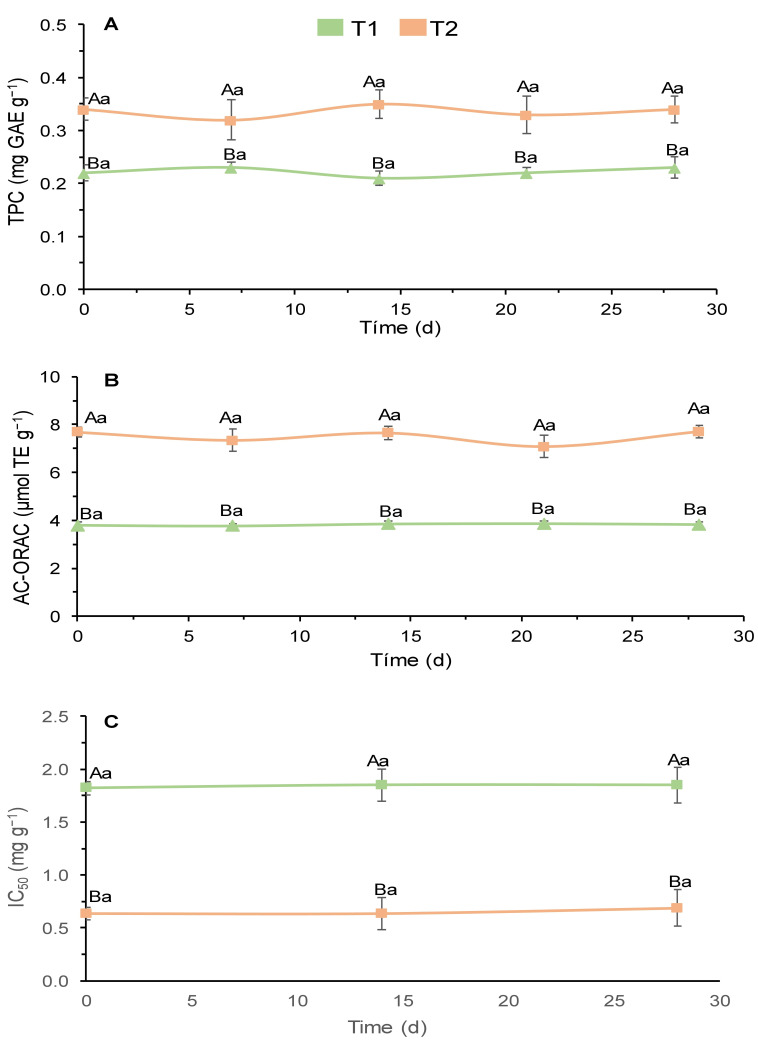
Total phenolic content (**A**), antioxidant capacity (**B**), and antihypertensive activity (IC_50_) (**C**) of the fermented SIC and YF mixture during storage at 4 °C. Different uppercase letters indicate significant differences between strains at the same storage time, whereas different lowercase letters indicate significant differences among storage times within the same strain (*p* < 0.05).

The two-way ANOVA revealed significant effects of treatment and storage time on TPC and AC (*p* < 0.05). According to Tukey’s test, T2 exhibited significantly higher TPC and AC values and lower IC₅₀ values compared to T1. For TPC, a significant interaction effect indicated that storage-related changes depended on the treatment applied. In contrast, AC and IC50 showed no significant interaction, suggesting similar storage trends across treatments. Although storage time had significant overall effects in the two-way ANOVA, one-way ANOVA within each treatment revealed no significant changes (*p* > 0.05) during the 28-day storage period. The average total phenolic content (TPC) of the SIC and YF mixture prior to fermentation was 0.24 ± 0.03 mg GAE mL^−1^ (Figure 7A). After fermentation, TPC values were 0.22 and 0.33 mg GAE mL^−1^ for T1 and T2, respectively. The absence of significant changes (*p* > 0.05) in TPC between the non-fermented sample and the fermented sample (T1) may be explained by the non-specific nature of the Folin–Ciocalteu assay, which reflects overall reducing capacity rather than individual phenolic compounds. During fermentation, phenolics may undergo simultaneous degradation, transformation, and release, leading to a dynamic equilibrium with no net variation. Fermentation can promote either increases or decreases in phenolic compounds, depending on enzymatic hydrolysis, matrix disruption, and extractability or, conversely, degradation, binding, and reduced accessibility [4]. Accordingly, these results indicate a limited effect of fermentation on TPC, in contrast with Adebo and Medina-Meza [4], who reported reductions using the same method.

These discrepancies may be attributed to differences in substrate composition, microbial strains, and fermentation conditions. The SIC-YF matrix may restrict phenolic accessibility or promote interactions with macromolecules, limiting detectable changes in reducing capacity. In addition, microbial metabolism can generate peptides and other reducing compounds that interfere with the Folin–Ciocalteu reaction. This is particularly relevant in T2, where simultaneous hydrolysis with Alcalase led to a 33% increase in TPC compared to T1, likely due to the formation of reducing peptides rather than a true increase in phenolics. Despite this difference, TPC remained stable during storage (*p* > 0.05), indicating minimal net changes over time in both treatments.

Previous studies have reported lower TPC values and a decline during storage. For instance, Luana et al. [39] reported 0.10–0.12 mg GAE mL^−1^ in fermented oat beverages, while Rai et al. [40] observed 0.18–0.40 mg GAE mL^−1^ in cereal-based yogurts, decreasing markedly after 28 days. In contrast, the stability observed in this study suggests greater preservation of reducing compounds, possibly due to matrix-specific interactions or protective effects of the system.

#### Comparative Phenolic Profile of the SIC–YF Mixture Before and After Fermentation

LC–MS/MS analysis (Table 2) revealed a complex and diverse phenolic composition, dominated by hydroxycinnamic acid derivatives, particularly caffeoylquinic acid isomers such as 5-O-caffeoylquinic acid (chlorogenic acid) and 4-O-caffeoylquinic acid (cryptochlorogenic acid), tentatively identified based on their characteristic fragmentation patterns, including diagnostic ions at *m*/*z* 191 and 173. Simple phenolic acids and feruloyl derivatives were also detected. 4,5-di-O-caffeoyl-2,7-anhydro-D-glycero-β-D-galacto-oct-2-ulopyranosonic acid (*m*/*z* 559) is a caffeic acid derivative found in yacon [41]. Similar phenolic profiles have been reported by Pacheco et al. [42] for yacon cultivated in Ecuador; however, the differences observed in the present study may be attributed to genotypic variability, as well as processing conditions, since the root was analyzed in dehydrated form and subjected to pasteurization at 90 °C.

The detection of p-coumaroyl diglucoside indicates that glycosylated precursors constitute a significant fraction of the phenolic pool. Higher molecular weight compounds tentatively assigned as ellagitannins were also detected. The flavonoid fraction consisted predominantly of glycosylated derivatives—kaempferol-3-O-rutinoside, rhoifolin, naringin, diosmin, hesperidin, and poncirin—identified through their corresponding aglycone fragments (*m*/*z* 285, 271, 299, and 301). Overall, the initial phenolic composition (Table 2) reflects a chemically complex matrix enriched in phenolic acids and flavonoid glycosides. While some similarities were observed with previous reports for yacon [42] and sacha inchi [43,44], notable differences were also evident, likely related to the distinct plant matrices analyzed (processed cake versus seed husk).

After fermentation with *L. rhamnosus* (T1), the majority of the original compounds were preserved, including chlorogenic acids, caffeic acid, feruloyl derivatives, ellagitannins, and flavonoid glycosides (Table 2). Minor modifications were observed, such as the appearance of putative adducts or conjugated species (e.g., *m*/*z* 723), along with slight variations in relative fragment intensities. These results indicate that *L. rhamnosus* exerts a moderate and selective impact on the phenolic matrix, favoring the retention of the original profile while promoting limited structural rearrangements. Compared with T2, the limited modifications observed in T1 suggest a lower biotransformation capacity of *L. rhamnosus* under the evaluated conditions. The preservation of key flavonoids such as naringin and hesperidin suggests that T1 maintains the antioxidant potential of the initial mixture, as supported by the relatively stable AC-ORAC values (Figure 7B).

In contrast, fermentation with *L. plantarum* (T2) resulted in a more extensive transformation of the phenolic composition (Table 2). This broader metabolite diversification was consistent with the higher antioxidant capacity and ACE inhibitory activity observed in T2. Several new metabolites were detected (*m*/*z* 702, 787, 614, 1175, 801), some of which could not be fully identified but exhibited fragmentation patterns consistent with galloyl-containing structures or naringin-derived conjugates. Concurrently, certain compounds present in the initial mixture, such as ellagitannins and rhoifolin, were no longer detected or appeared to be replaced by higher-molecular-weight derivatives. These modifications are also consistent with the reported ability of *L. plantarum* to metabolize hydroxycinnamic acids through reactions such as reduction, hydrolysis, and decarboxylation. Although specific metabolites such as dihydroferulate or vinylphenol derivatives were not conclusively identified, the observed changes in the phenolic profile suggest active phenolic bioconversion during fermentation.

The superior functional performance observed in T2 may be attributed to the synergistic interaction between Alcalase-mediated proteolysis and the metabolic activity of *L. plantarum*. Proteolysis likely increased the availability of peptides and amino acids, thereby stimulating microbial metabolism and the formation of bioactive metabolites. Similar findings were reported by [45], who demonstrated that protease supplementation enhanced microbial growth, enzyme secretion, sugar metabolism, and organic acid production during fermentation. In addition, microbial fermentation has been reported to improve the solubility and antioxidant potential of phenolic compounds through structural modification and enzymatic conversion [11]. Overall, these findings suggest that the combined effect of enzymatic hydrolysis and microbial metabolism contributed to the improved functional properties observed in T2.

This suggests that *L. plantarum* promotes more active enzymatic biotransformation, likely involving hydrolysis, deglycosylation, and subsequent recombination reactions, leading to the formation of novel conjugates and degradation products. Although chlorogenic acids and flavonoid glycosides were still detected, their altered fragmentation patterns indicate structural modifications relative to the initial state.

A comparison between T1 and T2 clearly demonstrates that *L. rhamnosus* fermentation is more conservative, largely maintaining the original phenolic fingerprint with only minor modifications, whereas *L. plantarum* induces more profound metabolic transformations, generating a broader diversity of phenolic metabolites. From a functional perspective, T1 may better preserve the intrinsic antioxidant properties of the raw materials, while T2 may promote the formation of new bioactive compounds with potentially enhanced or altered biological activities, which is consistent with the higher AC-ORAC values observed for T2 (Figure 7B). These findings highlight the importance of strain-specific metabolic activity in shaping the phenolic profile of fermented plant-based matrices and suggest that targeted fermentation strategies can be employed either to preserve or to diversify phytochemical composition, depending on the desired functional outcome.

The observed modifications in the phenolic profile following fermentation are primarily driven by the enzymatic machinery of LAB, which enables the hydrolysis of conjugated phenolics and the generation of structurally simpler and potentially more bioaccessible metabolites. Enzymes such as β-glucosidases, esterases, and phenolic acid decarboxylases have been widely reported to mediate the release and transformation of key phenolic acids, including caffeic and ferulic acids [11]. In addition, LAB fermentation has consistently been linked to enhanced total polyphenol content and antioxidant capacity across different food matrices, particularly those fermented with *L. rhamnosus* and *L. plantarum* [46]. The strain-dependent variations observed in this study further support previous evidence highlighting the superior phenolic biotransformation potential of *L. plantarum*, which is associated with the generation of a broader and more complex spectrum of phenolic metabolites [47].

### 3.6. Stability of Antioxidant and Antihypertensive Properties Throughout Storage

The results for ORAC AC and ACE IC_50_ are presented in Figure 7B,C. A clear difference between treatments T1 and T2 (both hydrolyzed with Alcalase) is observed in Figure 7B, with ORAC values approximately 50% lower in T1. A similar trend was reported by Campos et al. [9], who found that Alcalase-treated samples exhibited significantly higher AC, although the values reported here were comparatively lower.

In addition to the use of different strains (*L. rhamnosus* in T1 and *L. plantarum* in T2), this difference may also be explained by the higher polyphenol content and soluble protein levels observed in T2 (Figure 4A and Figure 7A). Soluble protein is associated with the generation of antioxidant peptides during protein hydrolysis, which are likely the main contributors to the enhanced AC in T2 [9]. In agreement, Zhao et al. [3] reported that AC is influenced by phytochemicals such as polyphenols, antioxidant polysaccharides, and bioactive peptides formed during fermentation, while Wongsa et al. [48] demonstrated that yogurt fortified with rice protein hydrolysates produced using Alcalase showed significantly increased AC.

During the 28-day storage period, no significant changes (*p* > 0.05) were observed in ORAC values, which ranged from 3.7 to 3.8 µmol TE g^−1^ for T1 and from 7.1 to 7.7 µmol TE g^−1^ for T2. This stability may be attributed to the consistent levels of total phenolic compounds and soluble protein—both key contributors to AC—throughout storage. Given that the SIC and YF mixtures in T1 and T2 are compositionally similar, comparable levels of phenolic antioxidants are expected; therefore, antioxidant peptides likely play a dominant role in ORAC values, and their stability explains the absence of significant variation over time.

Shori et al. [24] evaluated the AC of cashew milk yogurt co-cultured with different probiotic strains using DPPH and FRAP assays, reporting that AC, mainly linked to phenolic compounds, varied during storage depending on the strain and analytical method used. In comparison, Simsek et al. [49] reported ORAC values of 2.24 µmol TE mL^−1^ in fermented blends of germinated lentil and cowpea seeds mixed with vegetable juices.

Hydrolyzed SI protein produced using Alcalase has been shown to exhibit both strong antioxidant and antihypertensive properties, attributed to bioactive peptides generated during enzymatic hydrolysis that can scavenge free radicals and inhibit angiotensin-converting enzyme (ACE), thereby contributing to cardiovascular health [7,8]. ACE IC_50_ values averaged 0.66 and 1.84 mg g^−1^ for T1 and T2, respectively (Figure 7C), with the higher antihypertensive activity in T2 likely resulting from a greater production of ACE-inhibitory peptides during hydrolysis, consistent with previous reports.

Campos et al. [9] reported an IC_50_ of 0.89 mg g^−1^ for a treatment similar to T1 but using *L. plantarum* and 0.61 mg g^−1^ for a treatment comparable to T2. Similarly, Wu et al. [50] reported an IC_50_ of 0.42 mg mL^−1^—close to that obtained in this study—for whole oats subjected to solid-state fermentation with *L. plantarum* and *Rhizopus oryzae*. ACE IC_50_ values remained stable during storage in both treatments, likely due to the persistence of ACE-inhibitory compounds, including peptides derived from protein hydrolysis and phenolic compounds, which did not change significantly over time.

Overall, the superior functional performance observed in T2 compared to T1 appears to be associated with the synergistic interaction between Alcalase-mediated proteolysis and the metabolic activity of *L. plantarum*. Experimental evidence supporting this mechanism includes the higher soluble protein content, enhanced AC (ORAC), and lower IC_50_ values for ACE inhibitory activity observed in T2 from the beginning of storage. Previous studies have demonstrated that Alcalase hydrolysis of sacha inchi proteins generates bioactive peptides with antioxidant and antihypertensive activities [7], which may explain the improved functional properties observed in T2. In addition, LC–MS/MS analysis of the non-fermented mixture and fermented samples after 28 days of storage revealed greater diversity of phenolic metabolites in T2, suggesting more extensive phenolic biotransformation during fermentation. Although both treatments maintained stable biofunctional properties during storage, the combined hydrolysis–fermentation strategy applied in T2 generated a matrix with superior functional attributes that remained stable throughout refrigerated storage.

## 4. Conclusions

This study demonstrates that fermentation of a sacha inchi cake–yacon flour blend yields a stable multifunctional matrix with preserved probiotic viability, prebiotic potential, and bioactive properties during 28 days of refrigerated storage. Both treatments, namely fermentation with *Lactobacillus rhamnosus* (T1) and the simultaneous enzymatic hydrolysis (with protease) and fermentation by *Lactobacillus plantarum* (T2), maintained viable cell counts above the minimum threshold required for probiotic efficacy, confirming the suitability of this matrix as an effective carrier system. Distinct functional profiles were observed, with *L. rhamnosus* (T1) promoting higher microbial viability, while the combined hydrolysis–fermentation process (T2) enhanced metabolic activity and functional attributes, as evidenced by increased organic acid production, higher soluble protein content, and improved antioxidant and antihypertensive activities. Although VCC values were lower in T2, they remained within the recommended range for probiotic functionality. The stability of total phenolic content, antioxidant capacity, ACE inhibitory activity, and limited FOS degradation in both treatments indicates preservation of bioactive compounds and prebiotic functionality throughout refrigerated storage. Furthermore, strain-dependent effects were evident, with *L. plantarum* (T2) promoting greater phenolic transformation and functional enhancement, whereas *L. rhamnosus* (T1) largely preserved the original compositional profile. Overall, the integration of enzymatic hydrolysis and fermentation represents an effective strategy to tailor the functional properties of plant-based systems, highlighting the potential of agro-industrial by-products as sustainable sources of bioactive ingredients; however, further studies on sensory acceptance, in vivo validation, and scalability are required for industrial application.

## Figures and Tables

**Table 1:** Changes in soluble solids, total solids, and dietary fiber composition of fermented blends based on sacha inchi press cake and yacon flour during storage.

Parameter	T1 (Day 0)	T1 (Day 28)	T2 (Day 0)	T2 (Day 28)
Soluble solids (°Brix)	7.63 ± 0.18 ^a^	7.40 ± 0.16 ^a^	8.03 ± 0.17 ^a^	7.97 ± 0.11 ^a^
Total solids (%)	14.37 ± 0.15 ^a^	14.40 ± 0.41 ^a^	14.80 ± 1.11 ^a^	14.64 ± 1.25 ^a^
Soluble fiber (%)	0.87 ± 0.04 ^a^	0.89 ± 0.07 ^a^	0.87 ± 0.04 ^a^	0.99 ± 0.02 ^a^
Insoluble fiber (%)	2.61 ± 0.21 ^a^	2.35 ± 0.18 ^a^	2.45 ± 0.15 ^a^	2.60 ± 0.19 ^a^
Total dietary fiber (%)	3.39 ± 0.11 ^a^	3.37 ± 0.10 ^a^	3.32 ± 0.12 ^a^	3.59 ± 0.21 ^a^

Means (*n* = 3) followed by the same letter within the same row are not significantly different (*p* > 0.05).

**Table 2:** Tentative identification of phenolic compounds in the SIC–YF mixture before and after fermentation by QTOF-MS.

Peak N°	RT (min)	*m*/*z* [M−H]^−^	Error (ppm)	Formula	Fragments (Relative Abundance, %)	Tentative Identification ^a^
SIC-YF mixture before inoculation						
1	8.559	487.1438	−2.9	C_21_H_27_O_13_	487.1438 (100), 443.1901 (81.09), 193.0495 (28.89), 178.0260 (2.29)	Feruloyl derivative
2	8.696	487.1437	−3.1	C_21_H_27_O_13_	487.1437 (100), 193.0495(50.77), 443.1900 (4554), 178.0261 (7.00)	Feruloyl derivative
3	9.405	353.0876	0.8	C_16_H_17_O_9_	191.0557 (100), 353.0873 (20.97), 192.0591 (8.35), 293.1240 (5.54), 248.9849 (3.06)	5-O-caffeoylquinic acid (chlorogenic acid)
4	10.469	353.0873	0.0	C_16_H_17_O_9_	173.0451 (100), 353.0873 (19.45), 307.1228 (12.19), 281.1389 (8.60), 347.1173 (4.66), 237.1493 (4.26)	4-O-caffeoylquinic acid (cryptochlorogenic acid)
5	10.686	179.0347	1.7	C_9_H_7_O_4_	135.0451 (13.51)	Caffeic acid ^b^
6	15.342	533.0928	−0.6	C_24_H_21_O_14_	337.1494 (24.72), 343.1181 (2.08), 163.0391 (0.01)	p-Coumaroyl diglucoside
7	16.234	957.2886	7.2	C_49_H_49_O_20_	957.2886 (100), 193.0502 (14.03), 939.2772 (11.64), 487.1453 (11.33), 469.1348 (5.87), 940.2817 (5.24)	Ellagitannin
8	18.785	559.1100	2.1	C_26_H_23_O_14_	559.1100 (100), 161.0241 (12.33), 397.0772 (10.31), 235.0454 (10.0), 179.0347 (2.87), 203.0345 (1.52)	4,5-di-O-caffeoyl-2,7-anhydro-d-glycero-beta-d-galacto-oct-2-ulopyranosonic acid
9	19.289	593.1514	1.3	C_27_H_29_O_15_	593.1514 (100), 285.0399 (42.83), 286.0433 (4.28),	Kaempferol-3-O-rutinoside
10	20.284	577.1563	1.0	C_27_H_29_O_14_	271.0607 (100), 269.0452 (88.59), 577.1563 (57.17), 173.0448 (53.73), 270.0485 (12.31), 335.0763 (10.94)	Rhoifolin (Apigenin-7-O-neohesperidoside)
11	20.284	515.1198	1.6	C_25_H_23_O_12_	515.1198 (100), 353.0862 (39.41), 179.0339 (29.58), 173.0446 (28.06), 191.0550 (20.75), 209.0291 (16.72), 335.0760 (10.70)	3,4-di-O-caffeoylquinic acid
12	20.352	579.1716	0.3	C_27_H_31_O_14_	579.1716 (100), 271.0606 (95.89), 269.0451 (41.80), 577.1559 (31.88), 270.0485 (5.21), 151.0031 (1.10)	Naringin ^b^
13	20.49	515.1186	−0.8	C_25_H_23_O_12_	353.0867 (100), 191.0553 (30.05), 354.0899 (17.20), 375.0683 (14.21), 349.2007 (10.61)	Chlorogenic acid derivative
14	20.776	607.1667	0.7	C_28_H_31_O_15_	299.0554 (100), 607.1667 (79.93), 284.0318 (26.87), 503.2489 (18.58), 300.0587 (17.21), 183.0655 (2.63)	Diosmin
15	21.027	609.1824	0.8	C_28_H_33_O_15_	609.1824 (100), 301.0714 (88.36), 302.0747 (88.36), 303.0766 (2.37), 286.0482 (2.11), 325.0716 (1.77), 164.0112 (0.42)	Hesperidin ^b^
16	22.938	593.1881	1.9	C_28_H_33_O_14_	285.0761 (100), 593.1881 (80), 286.0792 (17.46), 309.0761 (0.73), 287.0819 (0.28), 164.0110 (0.12)	Poncirin
Treatment T1 after 28 days of storage						
2	8.679	487.1453	0.5		487.1453 (100), 443.1917 (75.17), 193.0502 (61.34), 178.0268 (7.40)	Feruloyl derivative
3	9.405	353.0871	−0.6	C_16_H_17_O_9_	191.0555 (100), 353.0869 (27.69), 293.1235 (11.93), 192.0588 (8.80), 179.0343 (3.43)	5-O-caffeoylquinic acid (chlorogenic acid)
4	10.469	353.0878	1.4	C_16_H_17_O_9_	173.0448 (100), 281.1381 (4.85), 307.1217 (4.45), 237.1485 (0.89)	4-O-caffeoylquinic acid (cryptochlorogenic acid)
5	10.721	179.0343	−0.6	C_9_H_7_O_4_	135.0446 (26.21)	Caffeic acid
6	15.273	533.0931	0.6	C_24_H_21_O_14_	533.0934 (100), 337.1500 (59.98), 399.2725 (29.28), 444.2467 (9.28), 163.0394 (8.63)	p-Coumaroyl diglucoside
7	16.206	957.2873	5.8	C_49_H_49_O_20_	957.2873 (100), 939.2767 (18.62), 193.0500 (9.52), 940.2794 (8.23), 487.1453 (6.84), 469.1342 (5.98), 899.2450 (4.70), 663.1923 (3.41)	Ellagitannin
8	18.785	559.1091	0.5	C_26_H_23_O_14_	559.1091 (100), 235.0453 (7.30), 161.0240 (5.94), 397.0769 (2.68), 293.0659 (1.08), 203.0343 (0.68), 236.0491 (0.51)	4,5-di-O-caffeoyl-2,7-anhydro-d-glycero-beta-d-galacto-oct-2-ulopyranosonic acid
9	19.243	593.1502	−0.7	C_27_H_29_O_15_	593.1502 (100), 285.0399 (45.33), 286.0437 (3.37), 401.1810 (2.55)	Kaempferol-3-O-rutinoside
10	20.284	577.1564	1.2	C_27_H_30_O_14_	271.0605 (100), 579.1713 (85.85), 269.0450 (76.66), 577.1559 (49.89), 580.1744 (22.64), 173.0449 (33.09)	Rhoifolin (Apigenin-7-O-neohesperidoside)
	20.284	515.1192	0.4	C_25_H_23_O_12_	515.1190 (100), 173.0450 (21.69), 353.0872 (6.89), 335.0764 (6.67)	3,4-di-O-dicaffeoylquinic acid
	20.352	579.1716	0.3	C_27_H_31_O_14_	271.0605 (100), 579.1716 (98.32), 269.0449 (40.20), 577.1560 (30.75), 559.1088 (23.21), 151.0031 (2.07)	Naringin
13	20.472	723.5016	−4.2		353.0858 (100), 191.0548 (46.07), 354.0894 (18.93), 375.0676 (18.25), 569.0366 (12.06), 677.7020 (9.89)	Chlorogenic acid derivative
14	20.776	607.1642	−3.5	C_28_H_31_O_15_	299.0546 (100), 607.1641 (76.94), 284.0314 (26.91), 503.2477 (21.51), 300.0580 (18.50), 285.0354 (4.01)	Diosmin
15	21.062	609.1808	−1.8	C_28_H_33_O_15_	301.0704 (100), 609.1802 (95.25), 325.0704 (3.59), 242.0574 (2.30), 164.0107 (0.91)	Hesperidin
16	22.937	593.1876	1.0	C_28_H_33_O_14_	285.0765 (100), 593.1876 (81.80), 286.0799 (17.47)	Poncirin
Treatment T2 after 28 days of storage						
1	8.559	702.3229			702.3229 (99.27), 675.3952 (32.26), 593.3419 (30.28), 487.1458 (14.37), 616.2614 (8.5), 466.1944 (0.18)	Unknown phenolic metabolite; absence of characteristic feruloyl fragment (*m*/*z* 193.0499)
2	8.662	487.1458			487.1458 (100), 193.0503 (61.97), 357.2504 (35.62), 443.1924 (20.40), 178.0267 (7.57), 214.1922 (4.11)	Feruloyl derivative
3	9.302	353.0873	−0.3	C_16_H_17_O_9_	191.0557 (100), 353.0872 (27.13), 192.0561 (9.01), 203.0823 (6.50)	5-O-caffeoylquinic acid (chlorogenic acid) ^b^
4	10.515	353.0870	−0.8	C_16_H_17_O_9_	173.0449 (12.95), 353.0870 (4.86), 257.1612 (4.12), 361.0957 (2.71), 470.3339 (2.30)	4-O-caffeoylquinic acid (cryptochlorogenic acid)
5	10.652	179.0341	−1.7	C_9_H_7_O_4_	179.0341 (100), 135.0443 (30.16)	Caffeic acid ^b^
6	15.273	614.3306			614.3306 (100), 570.3616 (23.31), 444.2458 (18.92), 414.2356 (8.03), 213.1237 (6.45), 533.0938 (5.84)	Unidentified phenolic compound; fragmentation suggests presence of galloyl group (loss 170 Da)
7	16.120	787.4675			787.4675 (100), 743.4410 (23.13), 474.2386 (11.99), 744.4439 (8.90), 745.4454 (2.07)	Unidentified high-molecular-weight phenolic metabolite; fragmentation not consistent with ellagitannins
8	18.819	559.1084	−0.7	C_26_H_23_O_14_	559.1084 (100), 161.0237 (7.90), 235.0449 (6.76), 397.0762 (6.60), 494.2028 (1.80), 362.2075 (1.36)	4,5-di-O-caffeoyl-2,7-anhydro-d-glycero-beta-d-galacto-oct-2-ulopyranosonic acid
9	19.208	593.3406			593.3420 (100), 493.2658 (57.62), 462.2346 (46.80), 484.2391 (24.21), 540.2452 (19.07), 285.0395 (1.0)	Kaempferol-3-O-rutinoside
11	20.284	515.1196	1.2	C_25_H_23_O_12_	515.1194 (89.43), 413.2765 (40.34), 444.2249 (27.17), 173.0450 (12.97), 353.0875 (8.01), 335.0773 (4.65)	3,4-di-O-Dicaffeoylquinic acid
12	20.352	1175.694			1175.6935 (100), 579.1725 (17.37), 271.0612 (15.52), 559.1102 (12.24), 677.4984 (8.35), 833.4530 (5.15)	Putative naringin-based conjugate; MS/MS fragments at *m*/*z* 579 (naringin) and 271 (naringenin)
13	20.490	801.3547			801.3542 (100), 717.3466 (22.30), 353.0879 (14.15), 617.4036 (13.53), 655.3162 (7.12), 191.0559 (3.01)	Chlorogenic acid derivative
14	20.81	607.1675	2.0	C_28_H_31_O_15_	486.2932 (100), 299.0558 (16.37), 284.0322 (4.50), 298.2491 (4.32), 456.2822 (4.23)	Diosmin
15	21.061	609.1816	−0.5	C_28_H_33_O_15_	609.1813 (100), 301.0708 (92.10), 302.0743 (16.96), 303.0763 (2.35), 286.0477 (1.95), 325.0710 (1.48)	Hesperidin
16	22.937	593.1873	0.5	C_28_H_33_O_14_	285.0759 (100), 593.1865 (81.36), 286.0791 (17.32), 309.0755 (0.52), 327.0871 (0.31)	Poncirin

^a^ Compounds were tentatively identified based on MS/MS fragmentation patterns, accurate mass measurements, and comparison with literature data. ^b^ Confirmed by comparison with an authentic standard.

## Data Availability

The original contributions presented in the study are included in the article/Supplementary Materials. Further inquiries can be directed to the corresponding author.

## Supplementary Materials

The following supporting information can be downloaded at: https://www.mdpi.com/article/10.3390/foods15122131/s1, Figure S1: Optimization of fermentation with Lactobacillus rhamnosus; Table S1: Proximate chemical composition of defatted sacha inchi cake (SIC) and yacon flour (YF); Table S2: Total sugar and FOS content in defatted sacha inchi cake and yacon flour; Table S3: Fiber content of defatted sacha inchi cake and yacon flour.
