# Supplementary material for: Storage Stability of a Multifunctional Fermented Blend Based on Sacha Inchi (Plukenetia volubilis) Oil Press Cake and Yacon (Smallanthus sonchifolius) Flour: Physicochemical Properties, Bioactivity, and Prebiotic–Probiotic Potential
Source: Foods. 2026 Jun 13;15(12):2131. doi: 10.3390/foods15122131 (PMC13298376; doi:10.3390/foods15122131)
Supplement: Supplementary file 1 [file foods-15-02131-s001.zip › foods-4301356-supplementary.pdf]

Supplementary Figure S1: Optimization of Fermentation with *Lactobacillus rhamnosus*

The optimization process was carried out under the conditions previously reported by [9]. Fermentation of the SIC and YF mixture was performed using *Lactobacillus rhamnosus* NRRL B-1937. The inoculum was prepared in 150 mL Erlenmeyer flasks containing 50 mL of MRS medium and incubated at 37 °C for 10 h in an orbital shaker at a constant speed of 125 rpm.

The fermentation process was conducted using a mixture of SIC and YF, each at a concentration of 7.5 %. A 60 mL portion of the mixture was transferred into 100 mL autoclavable flasks and subjected to ultrasound (US) pretreatment using a Branson Ultrasonics SFX250 system (Branson Ultrasonics Corporation, Danbury, USA) equipped with a 20 kHz converter and a ½″ microtip. The treatment was applied at 40 % amplitude for 5 min using 5 s on/off cycles. Subsequently, the mixture was pasteurized at 95 °C for 10 min, rapidly cooled, and inoculated with 1 % (v/v) of the prepared inoculum (7 Log10 CFU mL^{-1}).

Proximate chemical composition, soluble and insoluble fiber content, fructooligosaccharide (FOS) content, and sugar concentrations (glucose, fructose, and sucrose) were determined in SIC and YF. In the fermented samples, the following parameters were evaluated: pH, titratable acidity, viable cell count (VCC), lactic acid concentration, protein content, antioxidant capacity (ABTS and ORAC assays), and angiotensin-converting enzyme inhibitory activity (ACE-I).

To optimize the SIC and YF mixture composition, a 3-level, 2-factor factorial design (3^2) combined with response surface methodology (RSM) was employed. The experimental design included 12 treatments with three central points, and the total concentration of the SIC + YF mixture ranged from 10 % to 20 %. Multiple response variables were considered during optimization, including pH, titratable acidity, VCC, antioxidant capacity (ORAC), soluble protein content, and ACE-I activity. Experimental design and statistical analyses were performed using Statgraphics Centurion XV (StatPoint Technologies, Inc., Warrenton, VA, USA). All treatments were conducted in triplicate.

The optimization results are presented in the following figure.

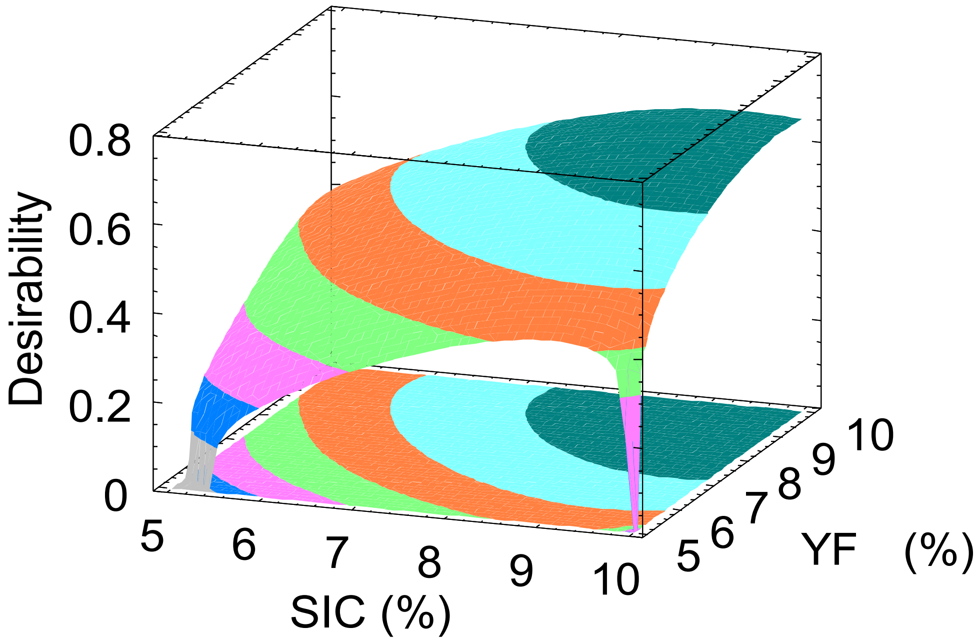

Desirability, 0.75

**Response**	**Optimum**
ACE inhibition (%)	88.99
Acidity (g lactic acid eq./100 g)	0.45
ORAC (µmol TE/g)	3.87
pH	4.41
Soluble protein (mg/g)	2.45
VCC (log, CFU/g)	9.99

Optimum concentration levels

SIC/9.5%. **YF/9.8%**

Supplementary Table S1. Proximate chemical composition of defatted sacha inchi cake (SIC) and yacon flour (YF)

Sample	Content (g/100 g)				
	Moisture	Fat	Protein	Carbohydrates*	Ash
SIC (%)	1.34 ± 0.05	4.58 ± 0.07	55.31 ± 1.93	32.59	6.18 ± 0.07
YF (%)	3.44 ± 0.11	0.48 ± 0.01	2.37 ± 0.05	89.04	4.49 ± 0.28

Values are expressed as the mean of three replicates ± standard deviation (SD).

*Carbohydrates were calculated by difference from the total (100%).

Supplementary Table S2. Total sugar and FOS content in defatted sacha inchi cake and yacon flour

Sample	Content (g/100 g)			
	Glucose	Fructose	Sucrose	FOS
SIC	ND	0.34 ± 0.04	5.81 ± 0.11	ND
YF	7.03 ± 0.16	20.51 ± 0.74	8.81 ± 0.20	32.95 ± 0.59

Values are expressed as the mean of three replicates ± standard deviation (SD). ND: not detected.

Supplementary Table S3. Fiber content of defatted sacha inchi cake and yacon flour

Sample	Content (g/100 g)		
	IDF	SDF	TDF
SIC	17.04 ± 0.19	3.36 ± 0.20	20.40 ± 0.01
YF	11.36 ± 0.33	3.32 ± 0.04	14.68 ± 0.36

Values are expressed as the mean of three replicates ± standard deviation (SD).
